# Electrospun PCL/PLA Scaffolds Are More Suitable Carriers of Placental Mesenchymal Stromal Cells Than Collagen/Elastin Scaffolds and Prevent Wound Contraction in a Mouse Model of Wound Healing

**DOI:** 10.3389/fbioe.2020.604123

**Published:** 2020-12-16

**Authors:** Eva Vonbrunn, Marc Mueller, Melanie Pichlsberger, Monika Sundl, Alexander Helmer, Stefanie Angela Wallner, Beate Rinner, Alexandru-Cristian Tuca, Lars-Peter Kamolz, Dagmar Brislinger, Birgit Glasmacher, Ingrid Lang-Olip

**Affiliations:** ^1^Division of Cell Biology, Histology and Embryology, Gottfried Schatz Research Center, Medical University of Graz, Graz, Austria; ^2^Institute of Multiphase Processes, Leibniz University Hanover, Hannover, Germany; ^3^Division of Plastic, Aesthetic and Reconstructive Surgery, Department of Surgery, Medical University of Graz, Graz, Austria; ^4^Division of Biomedical Research, Medical University of Graz, Graz, Austria; ^5^COREMED – Cooperative Centre for Regenerative Medicine, Joanneum Research Forschungsgesellschaft mbH, Graz, Austria

**Keywords:** Matriderm, PCL, PLA, electrospinning, wound healing, placenta, hAMSCs, human amnion-derived mesenchymal stem/stromal cell

## Abstract

Mesenchymal stem/stromal cells (MSCs) exert beneficial effects during wound healing, and cell-seeded scaffolds are a promising method of application. Here, we compared the suitability of a clinically used collagen/elastin scaffold (Matriderm) with an electrospun Poly(ε-caprolactone)/poly(l-lactide) (PCL/PLA) scaffold as carriers for human amnion-derived MSCs (hAMSCs). We created an epidermal-like PCL/PLA scaffold and evaluated its microstructural, mechanical, and functional properties. Sequential spinning of different PCL/PLA concentrations resulted in a wide-meshed layer designed for cell-seeding and a dense-meshed layer for apical protection. The Matriderm and PCL/PLA scaffolds then were seeded with hAMSCs, with or without Matrigel coating. The quantity and quality of the adherent cells were evaluated *in vitro*. The results showed that hAMSCs adhered to and infiltrated both scaffold types but on day 3, more cells were observed on PCL/PLA than on Matriderm. Apoptosis and proliferation rates were similar for all carriers except the coated Matriderm, where apoptotic cells were significantly enhanced. On day 8, the number of cells decreased on all carrier types except the coated Matriderm, which had consistently low cell numbers. Uncoated Matriderm had the highest percentage of proliferative cells and lowest apoptosis rate of all carrier types. Each carrier also was topically applied to skin wound sites in a mouse model and analyzed *in vivo* over 14 days via optical imaging and histological methods, which showed detectable hAMSCs on all carrier types on day 8. On day 14, all wounds exhibited newly formed epidermis, and all carriers were well-integrated into the underlying dermis and showing signs of degradation. However, only wounds treated with uncoated PCL/PLA maintained a round appearance with minimal contraction. Overall, the results support a 3-day *in vitro* culture of scaffolds with hAMSCs before wound application. The PCL/PLA scaffold showed higher cell adherence than Matriderm, and the effect of the Matrigel coating was negligible, as all carrier types maintained sufficient numbers of transplanted cells in the wound area. The anti-contractive effects of the PCL/PLA scaffold offer potential new therapeutic approaches to wound care.

## Introduction

Skin forms a protective shield against the environment (Zhao et al., 2016). When disrupted or injured, the skin heals via a complex and strictly regulated wound-healing process (Lazarus et al., 1994). Any deviations in this repair response can lead to chronic wounds, which are characterized by prolonged and sub-optimal inflammation, concurrent infection, deregulation of proteases (Eming et al., 2010), reduced growth factor activity (Lauer et al., 2000), stem cell dysfunction (Stojadinovic et al., 2014), and cellular senescence (Coppé et al., 2008). Cutaneous injury that penetrates into the dermis results in scarring and reduces skin function and quality (Hu et al., 2018a). Treating wounds and their complications often is associated with pain, including emotional and physical distress. Moreover, chronic wounds, deep, and extensive burns, and consecutive excessive scar formations are difficult to treat and constitute a large financial burden on the health care system.^1^ Therefore, new therapeutic approaches are highly warranted.

A promising approach in skin tissue engineering and cutaneous wound healing is the application of mesenchymal stem/stromal cells (MSCs) (Chen and Rogers, 2007). For example, perinatal tissue and its derivatives have clinical benefits in wound repair and regeneration (Silini et al., 2015; Pogozhykh et al., 2020). The human fetal amnion membrane has been used as a biological dressing for over 100 years. In the early 1900s, the amnion was used for skin transplantation (Davis, 1910) and for treating burns and skin ulcerations (Sabella, 1913; Stern, 1913). More recently, MSCs from fetal membranes have been transplanted without signs of immunological rejection, meaning that the application does not require immunosuppressive treatment (Ueta et al., 2002; Bailo et al., 2004; Jirsova and Jones, 2017). Human amnion-derived MSCs (hAMSCs) can be obtained non-invasively. These cells have anti-inflammatory, anti-cancer, and anti-fibrotic characteristics, and they are immunologically tolerated *in vivo* (Parolini et al., 2008; Silini et al., 2013). Additionally, hAMSCs secrete factors that are crucial for wound healing, such as epidermal growth factor I, IL-8, and IGF-1, which modulate migration and proliferation of keratinocytes, fibroblasts, and endothelial cells (Kim et al., 2012). Several studies confirm the angiogenic properties of hAMSCs *in vitro* and *in vivo* (König et al., 2012, 2015; Kinzer et al., 2014; Tuca et al., 2016; Ertl et al., 2018).

MSCs can be applied to wounds via intradermal injection or topical spraying, but these methods are associated with rapid MSC disappearance and death. Scaffold-based stem cell delivery has become increasingly popular. This method offers several advantages over other techniques, both in animal models of wound healing and in clinical settings, including complete wound coverage, protection of transplanted cells, preserved cell expression of stemness-related genes, and significantly accelerated wound healing (Xue et al., 2018; Mulholland, 2020).

Natural and synthetic biomaterials are used as carriers for transplanted cells. Natural biomaterials are widely used in reconstructive and burn injury treatments. For example, Matriderm is a bovine-derived collagen-elastin matrix used as an allogenic dermal substitute. However, these dermal substitutes have limitations, such as high cost, potential donor infection, and complicated surgical procedures (Halim et al., 2010; Al-Maawi et al., 2018). Synthetic biomaterials are low-cost alternatives that are precisely synthetized under controlled conditions to produce a biomaterial with specific porosity, thickness, and surface topography (Moore et al., 2001). For example, a promising material in wound repair is a blend of two biodegradable polymers, polycaprolactone (PCL) and poly-l-lactide acid (PLA). These materials offer a large surface for structural support of host cells and tissue regeneration.

The intention of this study was to evaluate these carrier types in terms of their ability to keep transplanted cells in the wound bed and improve healing outcomes. We hypothesized that synthetic electrospun carriers would outperform natural collagen/elastin ones. To test this hypothesis, we produced an epidermal-like electrospun PCL/PLA scaffold and assessed its function *in vitro* and *in vivo* using a mouse model of full-thickness wound healing. We then compared it with the dermal substitute Matriderm.

## Materials and Methods

### Production of PCL/PLA Scaffolds by Electrospinning

Polycaprolactone (PCL, Mn = 70,000–90,000, Sigma-Aldrich) and poly-l-lactide acid (PLA, Mw = 150,000, Natureplast) were dissolved in 2,2,2-trifluoroethanol (Sigma-Aldrich) at concentrations of c_PCL_ = 100 mg/ml and c_PLA_ = 50 mg/ml for the apical layer and c_PCL_ = 200 mg/ml and c_PLA_ = 100 mg/ml for the basal layer. Polymer blends were kept on a stirrer overnight to ensure homogenous solutions. Two-layer fiber mats (PCL/PLA 100/50 and PCL/PLA 200/100) were fabricated on a customized electrospinning machine (Zernetsch et al., 2016). A syringe pump fitted with a blunt needle applied a constant flow rate of 3 ml/h (diameter = 0.8 mm). The distance between the needle and rotating drum collector (diameter = 150 mm) was kept at 250 mm, and the applied voltage was 25 kV. Each layer was spun at 250 rpm for 1 h.

### Material Characterization of PCL/PLA Scaffolds and Collagen-Elastin Scaffolds

Cryo-sections of the PCL/PLA and collagen-elastin scaffolds (Matriderm, Medskin Solutions) were prepared to analyze the configuration and wall thickness of the samples. The fiber morphology of the scaffolds was characterized using scanning electron microscopy (VP-SEM S3400, Hitachi Europe). Six samples were taken from each fiber mat and coated with gold palladium (Sputter Coater SC7620, Emetich) for 45 s. Images were acquired and analyzed according to a standard operating procedure (Fuchs et al., 2019). Fiber diameter distribution and mass of samples were used to calculate the specific surface of the electrospun scaffolds using the following equations:

$$l_{f}=\frac{m_{f}}{\rho \pi \sum_{i=0}^{x}{\left(\frac{d_{x}}{2}\right)}^{2}\;h_{x}}$$

$$\begin{array}{l} A_{f}=l_{f}\overset{x}{\underset{i=0}{\sum }}\pi d_{x}h_{x} \end{array}$$

$$\begin{array}{l} S_{m}=\frac{A_{f}}{m_{f}} \end{array}$$

where *l*_*f*_ = length of the endless fiber; *m*_*f*_ = mass of the endless fiber; ρ = density of the polymer; *d*_*x*_ = diameter of the volume segment; *h*_*x*_ = frequency of the fiber diameter; *Af* = skin surface; and *Sm* = specific surface.

The wettability of the scaffolds was analyzed using the captive bubble technique, which can assess contact angles of porous or rough materials. Samples (10 × 40 mm) were immersed in Dulbecco's Modified Eagle's Medium. An air bubble was placed at the bottom of the sample, and the contact angle was assessed using an EasyDrop goniometer (Krüss).

Thermal properties of the electrospun scaffolds were assessed using differential scanning calorimetry (DSC 204 F1 Phoenix). Samples of 5–12 mg were analyzed at a heating rate of 10 K/min and cooling rate of 20 K/min. Measurements were conducted until reaching 50°C above the assumed melting point and 50°C below the assumed glass transition temperature.

### Cell Characterization and Seeding of the Scaffolds With hAMSCs *in vitro*

The ethical committee of the Medical University of Graz approved the human study (No. 21-079 ex 09/10). All participants provided written informed consent for the scientific use of placental tissue. We collected human term placentas from women with normal pregnancies immediately after birth (38–42 weeks). The hAMSCs then were isolated from the amnion according to the protocol of Soncini et al. (2007) and as described in König et al. (2012) and König et al. (2015).

Specifically, the amnion and chorion were manually separated and washed with 0.9% saline (Fresenius Kabi) supplemented with 150 IU/mL penicillin, 150 mg/mL streptomycin (both from PAA Laboratories), and 0.4 mg/mL amphotericin B (Gibco, Invitrogen). The amnion was cut into small pieces and incubated with 2.5 U/mL dispase (BD Biosciences) for 9 min at 37°C. The pieces were transferred into low-glucose Dulbecco's Modified Eagle's Medium (Gibco, Invitrogen) supplemented with 15% fetal bovine serum gold (Gibco, Invitrogen), 100 IU/mL penicillin, and 100 mg/mL streptomycin for 10 min. Subsequently, 1.0 mg/mL of collagenase A and 0.01 mg/mL of DNase were added for 2 h (both from Roche). After centrifugation for 3 min at 150 g, the supernatant was poured through a cell strainer (100-mm mesh size; BD Biosciences) and centrifuged for 10 min at 300 g. The pellet was washed with phosphate-buffered saline (Gibco, Invitrogen) and resuspended in 10 mL of endothelial growth medium-2 (EGM-2; Lonza) containing 2% fetal bovine serum, epidermal growth factor, hydrocortisone, vascular endothelial growth factor, basic fibroblast growth factor, insulin-like growth factor 1, ascorbic acid, and heparin. After further centrifugation for 10 min at 300 g, the cell pellet was resuspended in 10 mL of EGM-2, and cells were cultured on 1% gelatin-coated flasks (PAN-Biotech). The medium was changed every 2–3 days.

The hAMSCs were cultured in EGM-2 (PromoCell), as previously described (König et al., 2012, 2015). We used only well-characterized hAMSCs at passages 3–5. The hAMSCs were positive for CD90, CD73, CD105, HLA-ABC, CD146, CD63, CD29, CD166, CD13, CD10, and CD49a and negative for immune and endothelial markers CD45, HLA-DR, CD14, CD3, CD19, CD15, and CD31. In addition, they lacked expression of CD271, alkaline phosphatase, and mesenchymal stem-cell like antigen-1. The hAMSCs also showed osteogenic and adipogenic differentiation potential.

To prepare the carriers, circular pieces (diameter = 8 mm) of PCL/PLA and Matriderm were die-cut using a biopsy punch and fixed at the bottom of a 12-well plate with a drop (10 μl) of EGM-2. Matriderm and the wide-meshed layer of PCL/PLA were seeded with hAMSCs (5 × 10^5^ cells) which were suspended in 50 μl of EGM-2 (uncoated carriers) or Matrigel (Corning) (coated carriers). The carrier membranes were then cultured at 37°C for 3 days or 8 days.

### Immunohistochemistry of Cell-Seeded Scaffolds *in vitro*

The carriers were fixed in 4% formalin for 1 h, cut in half, and washed with phosphate-buffered saline. Scaffolds were automatized, dehydrated, and processed in standard paraffin wax (Vogel) using a Tissue-Tek VIP^®^ Vacuum Infiltration Processor (Sakura) and the TES Valida Dispenser Unit (MEDITE), as described in Table 1. Serial sections (8–10 μm) were taken from the scaffolds at 100 μm intervals. Slides were deparaffinized, and different antigen retrievals were tested to find the ideal method for antigen-demasking before the immunostaining.

**Table 1:** Protocol for the automatized dehydration and paraffinization with the tissue-Tek^®^ VIP^®^ vacuum infiltration processor.

	**Time (min)**	**Temperature (^**°**^C)**
60% ethanol	60	40
80% ethanol	60	40
96% ethanol	60	40
100% ethanol	60	40
100% ethanol	60	40
100% ethanol	60	40
Tissue clear	60	40
Tissue clear	60	40
Tissue clear	60	40
Paraffin	60	56
Paraffin	60	56
Paraffin	60	56

Low-temperature enzymatic antigen demasking was performed using pepsin, hyaluronidase, and proteinase K. Slides were either incubated with pepsin (0.1% in diethylpyrocarbonate; Sigma-Aldrich) for 30 min, hyaluronidase (0.02% in phosphate-buffered saline and 0.01% bovine serum albumin; Sigma-Aldrich) for 20 min, or proteinase K (5% in TE-Buffer pH 8; Roche) for 15 min at 37°C. Alternatively, heat-induced antigen retrieval was performed in a KOS microwave (Milestone) with Target Retrieval Solution pH 9 (Dako) at 93°C for 15 min. After blocking in H2O2 Block (ThermoScientific) for 10 min and UV Block (ThermoScientific) for 5 min, slides were exposed to anti-vimentin (0.078 μg/ml; Dako) for 30 min to detect hAMSCs on the carriers. Slides were developed using the UltraVision LP Detection System (ThermoScientific).

### Double-Fluorescence and Quantitative Analyses of Cell-Seeded Scaffolds *in vitro*

For each carrier type, three sections at 100 μm apart were stained via immunofluorescence. Antigen retrieval was performed in the KOS microwave at pH 6 and 93°C. After blocking in UV Block (ThermoScientific) for 5 min, anti-Ki-67 (1.1 μg/ml; Dako) and anti-Caspase 8 (1:100, Cell Signaling Technology) were applied for 30 min to detect respective proliferating and apoptotic cells. For fluorescence staining, secondary antibodies with Alexa Fluor dyes (1:400; Invitrogen) were applied for 30 min. Nuclei were stained for 5 min with DAPI (1:2000; Life Technologies), and the slides were permanently covered with ProLong Gold (Life Technologies).

For image acquisition and analysis, we used a Zeiss Observer.Z1 inverted microscope. We acquired 8–10 images of each slide and then made adjustments using ZEN 2 blue software version 2.0.0.04.8.2.0 (Zeiss). Wavelength and exposure time were determined with auto exposure and set for all pictures for Ki-67 (tetramethyl rhodamine isothiocyanate at 532 nm; 180 ms), Caspase 8 (fluorescein isothiocyanate at 495 nm; 80 ms), and DAPI (358 nm; 230 ms). The cell image analysis software CellProfiler was used to count proliferating cells, apoptotic cells, and all nuclei. To identify the number of nuclei, a pipeline was designed using the software's Identify Primary Object option, determined by the signal intensity and size. For quantification of proliferating and apoptotic cells, the Identify Secondary Object was used, and only fluorescein isothiocyanate and tetramethyl rhodamine isothiocyanate signals in connection with DAPI signals were counted.

The total number of hAMSCs on the slides was quantified and averaged for each carrier type. Cell quality was compared by using the ratio of proliferating and apoptotic cells in relation to the total cell number. Statistical analysis was conducted with GraphPad Prism 8 software. The percentage of proliferating or apoptotic cells on each carrier type was tested for normal distribution using the Kolmogorov-Smirnov test and the Shapiro-Wilk test with a *p*-value of 0.05. As data were not normally distributed in either case, the non-parametric Kruskal-Wallis test for independent samples (*p* < 0.05) was conducted. If significant differences occurred for proliferation or apoptosis, a *post-hoc* test with the non-parametric Mann-Whitney *U*-test for multiple comparisons was used to compare the different carriers. The *p*-value thus had to be adapted via the Bonferroni correction, which meant dividing the primary *p*-value of 0.05 by the number of carriers, resulting in *p* < 0.0125.

### Mouse Model of Wound Healing

The ethics commission of the Animal Care and Use Committee in Vienna approved the animal experiment. Three days prior to injury, hAMSCs were fluorescence-labeled via 30 min of incubation at 37°C with CellTracker™ Green CMFDA (10 μM; Life Technologies). The labeled hAMSCs then were suspended in 50 μl of EGM-2 or Matrigel, seeded onto Matriderm or the wide-meshed layer of PCL/PLA (5 × 10^5^ cells per carrier), and cultured *in vitro* before applying them to the animal wounds. Matriderm and PCL/PLA carriers without cells were similarly processed and served as negative controls.

One flask with labeled cells was harvested, pelleted, and observed in the CRi Maestro imaging system to study the staining intensity *in vivo*. Additional carriers were seeded with the labeled hAMSCs to investigate the efficiency of the fluorescence staining *in vitro*. After 3 days of incubation, the carriers were fixed in 4% formaldehyde for 1 h, washed in phosphate-buffered saline, and incubated with DAPI for 5 min. After washing and drying, the carriers were permanently covered with ProLong Gold and analyzed with a laser scanning microscope (Leica Microsystems).

We created two full-thickness wounds (8-mm diameter) using a circular-punch biopsy on the dorsal side of 10 female NMRI-Foxn1^nu^/Foxn1^nu^ nude mice (Janvier Labs). All mice received anesthesia and analgesia. To evaluate treated wound healing, the wound pairs of four mice were treated immediately with cell-seeded carriers and their corresponding controls as follows: one received uncoated Matriderm ± hAMSCs, one received Matriderm coated with Matrigel ± hAMSCs, one received uncoated PCL/PLA ± hAMSCs, and one received PCL/PLA coated with Matrigel ± hAMSCs. It is important to note that we applied the two-layered PCL/PLA scaffold on the wounds so that the wide-meshed (cell-populated) layer faced the wound bed and the dense-meshed (cell-free) layer served as apical protection. To evaluate untreated wound healing, the wound pairs of four additional mice received no treatment (no carriers, no hAMSCs) except Tegaderm (3M). Another mouse received uncoated Matriderm without hAMSCs for both wounds, and another mouse received uncoated PCL/PLA without hAMSCs for both wounds. All wounds were covered with Tegaderm (3M). Directly after the surgical procedure, all mice received a single-shot antibiotic. All mice also received postoperative analgesia and were housed in groups.

Wounds of hAMSC-treated mice were monitored using the CRi Maestro *in vivo* imaging system (445–490 nm excitation and 515 nm long-pass emission) on the day of injury (day 1) and then on days 3, 8, and 14. Two additional mice with no wounds served as references for monitoring the *in vivo* imaging system.

### Evidence of Carriers in Wound Tissue and Immunohistochemical Analysis

All mice were sacrificed after 14 days. Their wounds were photographed, and the wound areas were excised, fixed in 4% formalin, embedded in paraffin, and sectioned at 100–200 μm intervals. Five serial sections (5 μm) were collected up to the maximal wound area to perform H&E staining. Sections of interest were further processed for immunohistochemistry. Antigen retrieval was performed at pH 6. Rat-anti-mouse CD31 (2 μg/mL, 1 h, Dianova) was used to identify murine blood vessels. Staining was visualized using a biotinylated rabbit-anti-rat antibody (10 μg/mL, VectorLab) and streptavidin-peroxidase reagent (10 min; ThermoScientific) after blocking with H2O2 (10 min), avidin and biotin block (both 15 min; VectorLab), and protein block (10 min). To detect hAMSCs, we performed antigen retrieval at pH 9. After blocking in H2O2 (10 min; ThermoScientific), UV block (5 min; ThermoScientific), M.O.M™ (1 h; VectorLab), and protein block (30 min; Dako), slides were exposed to anti-vimentin for 30 min (0.078 μg/ml; Dako) and developed using the UltraVision LP Detection System (ThermoScientific). Alternatively, we used anti Ki-67 (1.1 μg/ml; Dako) to detect proliferating hAMSCs and goat-anti mouse fluorescein isothiocyanate (BD) as a secondary antibody.

## Results

### Material Characterization of PCL/PLA and Matriderm

**Figure 1:**
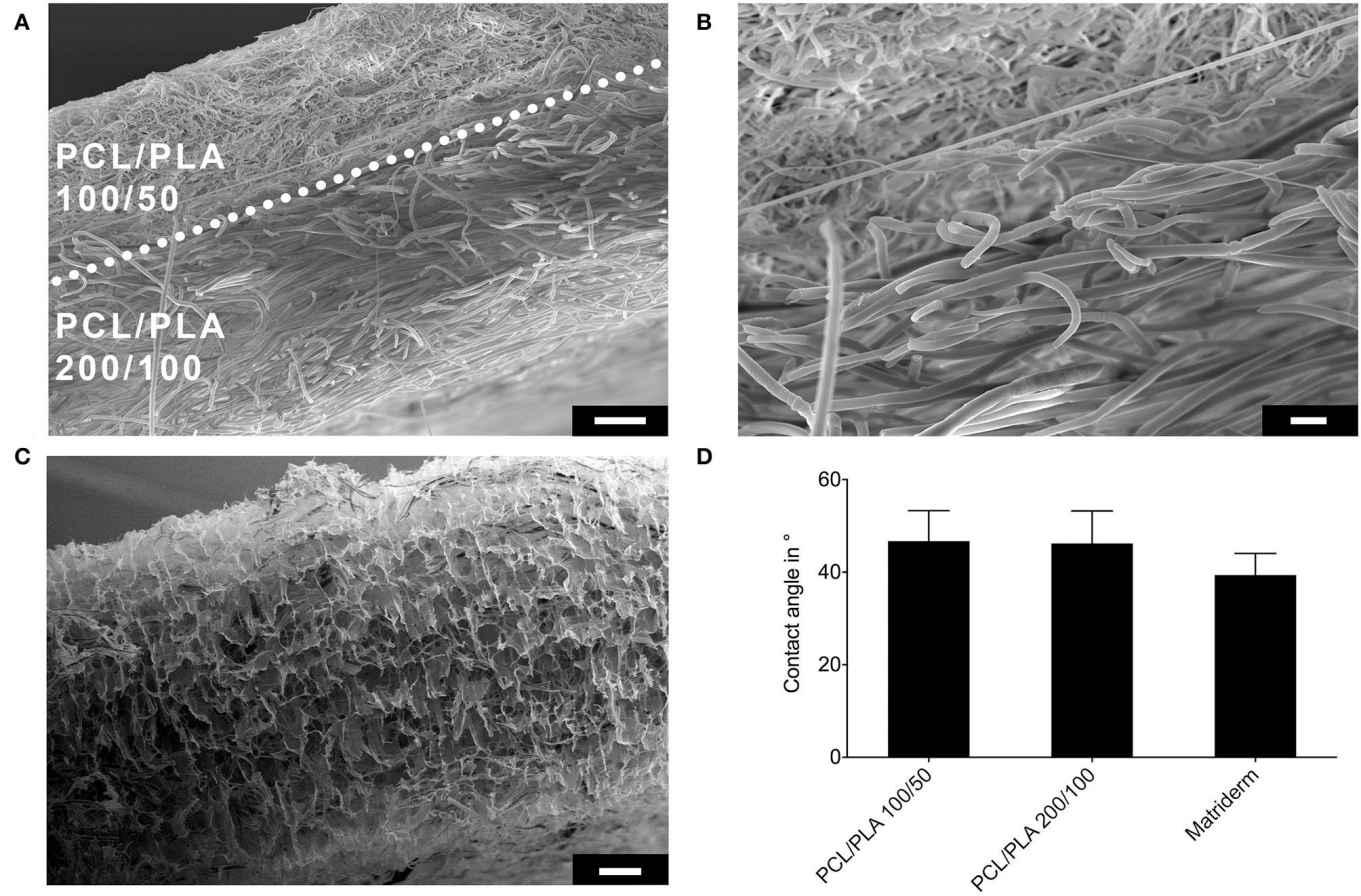
Evaluation of wall structure and wettability of PCL/PLA and Matriderm scaffolds. **(A)** Scanning electron microscope image of a cross-section of the two-layered PCL/PLA scaffold. Dotted line marks the border between the two layers. Scale bar = 20 μm. **(B)** High-magnification image of the region shown in A (scale bar = 10 μm). **(C)** Scanning electron microscope image of a cross-section of Matriderm. Scale bar = 100 μm. **(D)** Contact angles of different scaffold materials to cell culture medium (*n* = 10–12).

Sequential electrospinning of PCL/PLA blends resulted in two-layered fibrous scaffolds without signs of delamination or bead deposits (Figure 1A). Structural examination revealed that both layers differed in their microstructure. The PCL/PLA 100/50 layer had a wall thickness of 82.4 ± 3.6 μm and consisted of densely packed small fibers (dense-meshed layer). The PCL/PLA 200/100 layer had a wall thickness of 203 ± 8.9 μm and consisted of loosely packed larger fibers (wide-meshed layer) (Figure 1B). Matriderm had a wall thickness of 804.3 ± 49.1 μm and a sponge-like microstructure (Figure 1C). Contact angle measurements were performed to reveal the wettability properties of the scaffolds. Differences in the fiber microstructure did not influence the wettability of the electrospun layers. Both PCL/PLA 100/50 and PCL/PLA 200/100 showed contact angles of 46 ± 7° to cell culture medium. Matriderm tended to show a narrower contact angle of 39 ± 5° (Figure 1D).

**Figure 2:**
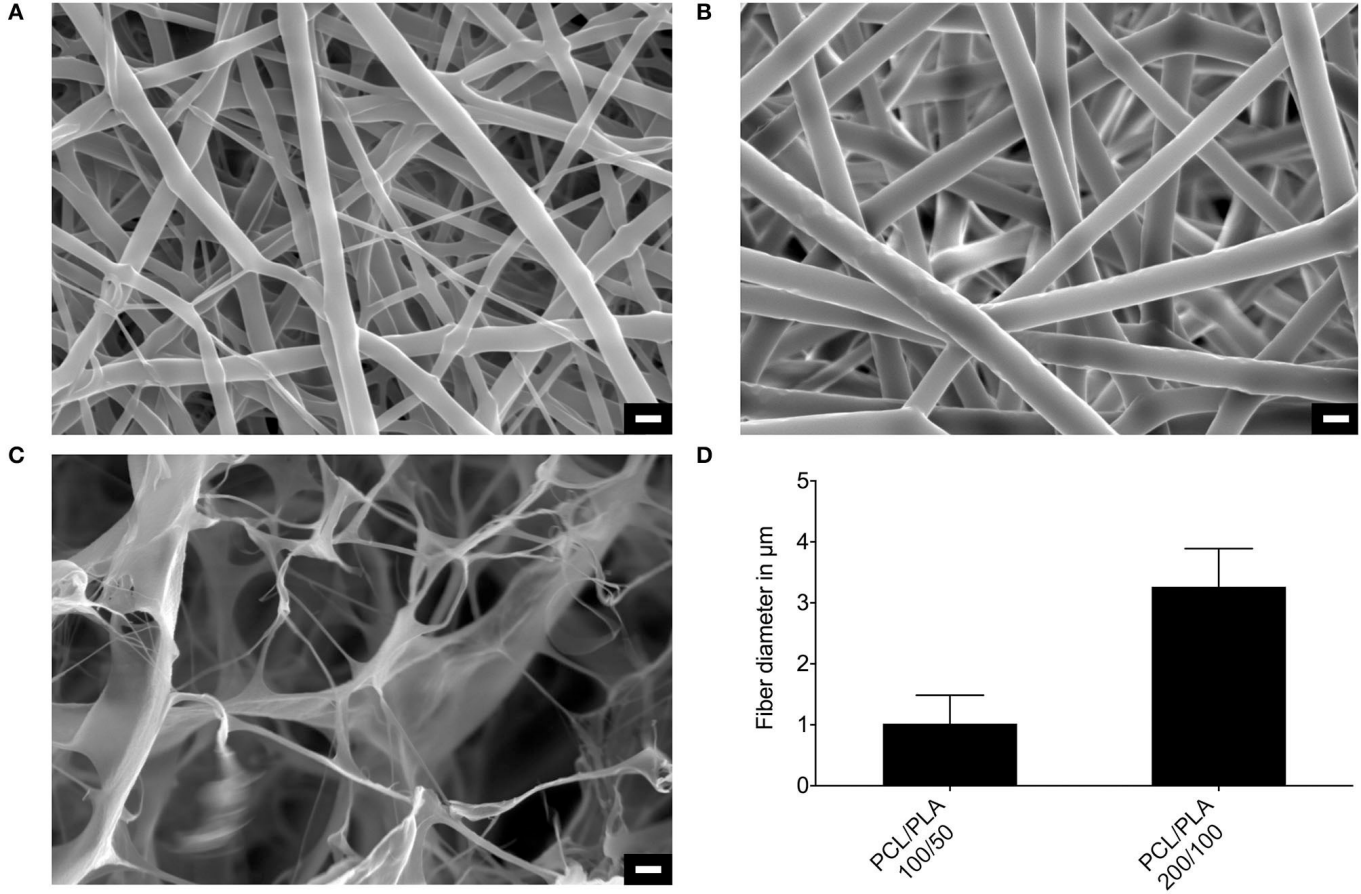
Microstructure of PCL/PLA scaffolds and Matriderm. Scanning electron microscope images of **(A)** PCL/PLA 100/50, **(B)** PCL/PLA 200/100, and **(C)** Matriderm. **(D)** Morphometrical analysis of the fiber thickness of the PCL/PLA material (*n* = 600). Scale bar = 3 μm.

Dense-meshed PCL/PLA 100/50 blends showed homogenous fiber-based structures with mean diameters of 1 ± 0.5 μm (Figures 2A,D). Higher blend concentrations led to homogeneous fibers with increased diameters (3.3 ± 0.6 μm) in the wide-meshed PCL/PLA 200/100 layer (Figures 2B,D). Matriderm had a highly porous sponge-like microstructure consisting of thin walls and fibers of about 0.6 μm (Figure 2C). The surface area of PCL/PLA 200/100 was smaller than that of PCL/PLA 100/50 (0.99 vs. 2.8 m^2^/g).

Thermograms indicated individual melting points of 57°C for PCL and 169.6°C for PLA. The determination of melting points for the PCL/PLA blends was not possible, since peaks for the glass transition temperature of PLA and melting point of PCL overlapped (data not shown).

### Antigen Retrieval and Immunostaining

**Figure 3:**
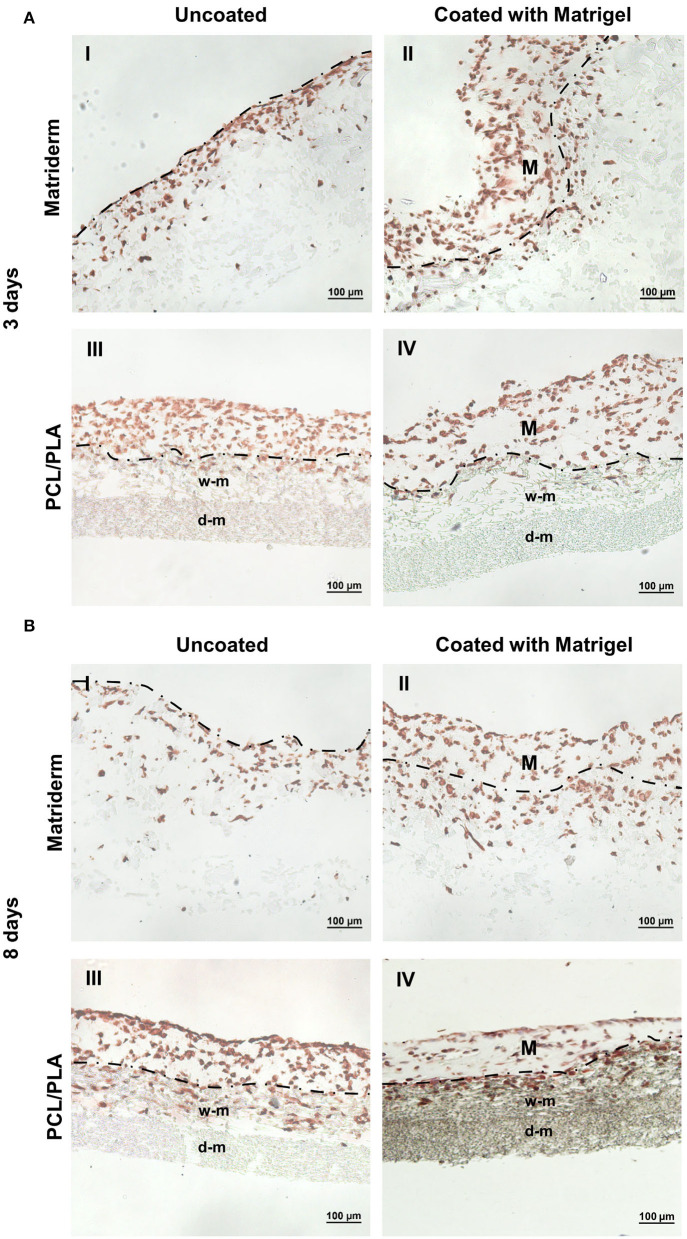
Immunohistochemical staining of hAMSC-seeded carriers to detect cell adhesion and migration. Matriderm and PCL/PLA were cultured with hAMSCs suspended in EGM-2 (uncoated) or Matrigel (coated) for **(A)** 3 days and **(B)** 8 days. The hAMSCs were visualized with anti-vimentin (brown color). The dotted line marks the side of Matriderm and PCL/PLA that was seeded with cells. Matrigel (M) was applied to the surface of the coated Matriderm and PCL/PLA layers. w-m, wide-meshed PCL/PLA layer; d-m, dense-meshed PCL/PLA layer.

**Figure 4:**
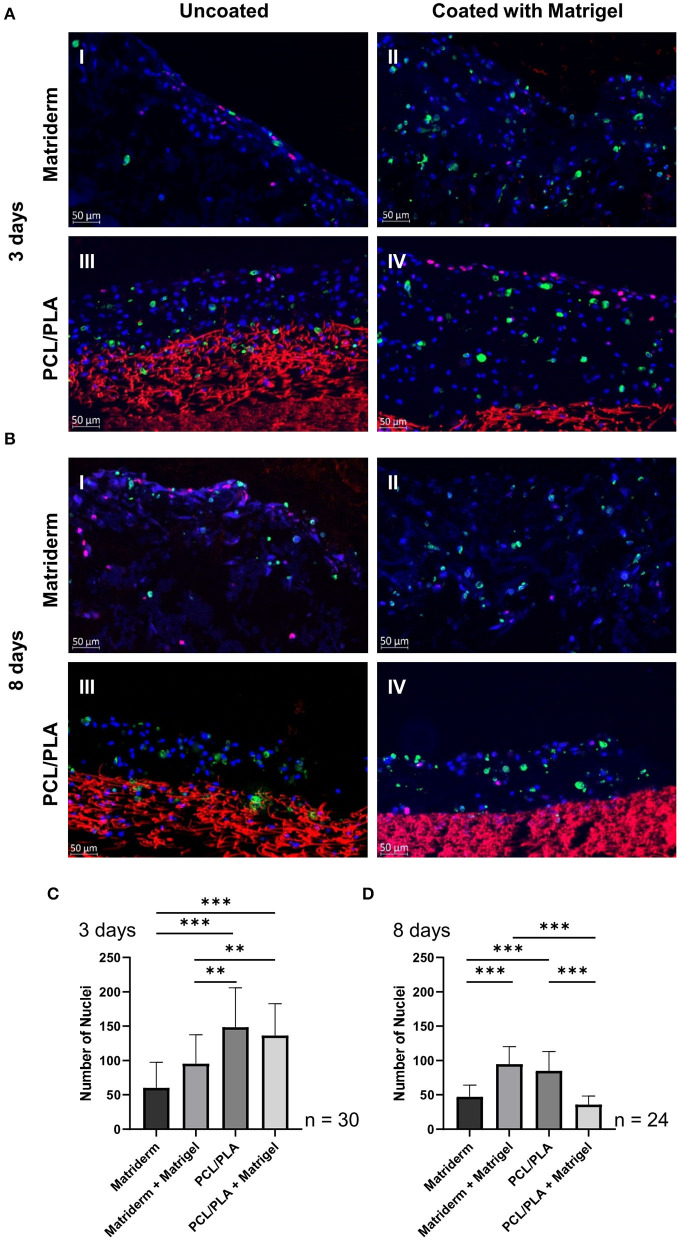
Immunofluorescent staining of hAMSC-seeded carriers to detect proliferating and apoptotic cells. Matriderm and PCL/PLA were cultured with hAMSCs suspended in EGM-2 (uncoated) or Matrigel (coated) for **(A)** 3 days and **(B)** 8 days and stained with DAPI (blue), anti-Ki67 (pink), and anti-caspase 8 (green) to detect cell nuclei, proliferating cells, and apoptotic cells, respectively. PCL/PLA showed a distinct red autofluorescence. The total cells on the cell-seeded carriers were assessed after **(C)** 3 days and **(D)** 8 days. Data are means ± SDs of three different slides per carrier type, cell count of 30 pictures per carrier. ***p ≤ 0.001, **p ≤ 0.01.

Enzymatic antigen demasking with pepsin, hyaluronidase, and proteinase K did not yield the desired results because the immunohistochemical staining did not work under these conditions. Additionally, the treatment with pepsin led to digestion of the Matriderm membrane (data not shown). Antigen retrieval under heat exposure (93°C) in the KOS microwave at pH 9 and pH 6 led to successful staining results with anti-vimentin (Figure 3), anti-Ki-67, and anti-Caspase 8 (Figure 4). The PCL/PLA and Matriderm scaffolds both preserved their morphological structure despite the heat exposure.

### hAMSC Attachment and Infiltration Into the Scaffolds *in vitro*

The hAMSCs colonized on both Matriderm and PCL/PLA with or without Matrigel coating. On day 3, the uncoated Matriderm had the fewest cells attached: hAMSCs invaded the first third of the Matriderm membrane with some cells migrating through the whole scaffold (Figure 3AI). The coated Matriderm contained a thick layer of evenly distributed cells (Figure 3AII). On the uncoated PCL/PLA, hAMSCs mainly covered the surface of the wide-meshed layer: few cells were visible in the wide-meshed layer, and no cells were observed in the fine-meshed layer (Figure 3AIII). The coated PCL/PLA contained a thick layer of evenly distributed cells (Figure 3AIV). In the Matrigel-coated scaffolds, single hAMSCs migrated into the superficial part of the scaffolds but mainly stayed within the Matrigel matrix.

On day 8, the hAMSCs migrated deep into and colonized large parts of the Matriderm scaffold (Figures 3BI,II). The Matriderm carriers also appeared soaked and slightly tattered. The hAMSCs migrated deep into the wide-meshed layer of the PCL/PLA scaffold, but no cells were observed in the fine-meshed layer (Figures 3BIII,IV). The coated Matriderm (Figure 3BII) and uncoated PCL/PLA (Figure 3BIII) had more hAMSCs than the uncoated Matriderm (Figure 3BI) or coated PCL/PLA (Figure 3BIV).

### Quantitative and Qualitative Evaluation of hAMSC-Seeded Scaffolds

To evaluate the quantity and quality of hAMSCs on the different materials, the cell-seeded carriers were fluorescence-stained with DAPI, anti-Ki67, and anti-Caspase 8 to detect cell nuclei, proliferating cells, and apoptotic cells, respectively. The PCL/PLA material showed a strong red background fluorescence, which was excluded in the software-based assessment. Proliferating cells were located mostly on the apical surface of the carriers or on top of the cell layer, whereas apoptotic cells were more evenly distributed throughout the whole cell matrix (Figures 4A,B).

For quantitative analysis, the total cells on each carrier type were counted. On day 3, the uncoated PCL/PLA had the highest number of attached cells (148.7 ± 10.4 hAMSCs), followed by the coated PCL/PLA (136.6 ± 8.5 hAMSCs). Both PCL/PLA carriers had significantly more cells than the Matriderm carriers (*p* = 0.0–0.006). The fewest cells were counted on uncoated Matriderm (60.3 ± 6.8 hAMSCs) (Figure 4C). On day 8, the number of cells decreased on all carrier types except the coated Matriderm (95.5 ± 7.7 hAMSCs). Slightly fewer cells were detected on uncoated PCL/PLA (84.9 ± 5.7 hAMSCs). The uncoated Matriderm (47.3 ± 3.5 hAMSCs) and the coated PCL/PLA (35.8 ± 2.6 hAMSCs) had significantly fewer cells (*p* < 0.0001) on day 8 (Figure 4D).

**Figure 5:**
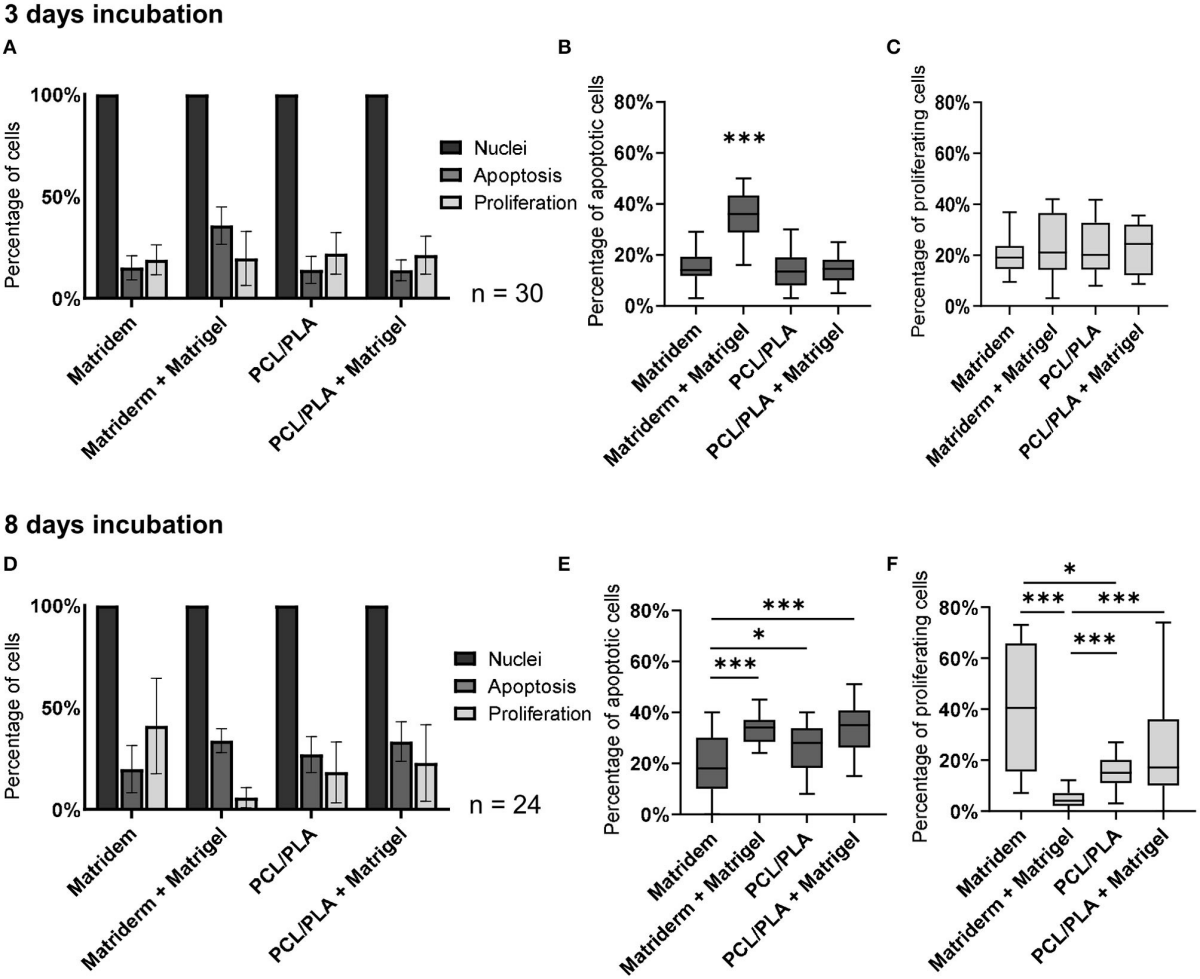
Percentage of proliferating and apoptotic hAMSCs in cell-seeded carriers. The number of proliferative and apoptotic cells in relation to the total cell number was evaluated on carriers cultured for **(A–C)** 3 days and **(D–F)** 8 days. ***p ≤ 0.001, *p ≤ 0.05.

To compare the quality of cells cultured on coated and uncoated PCL/PLA and Matriderm, we estimated the ratio of proliferating and apoptotic cells to the total number of cells. On day 3, the apoptosis ratio was significantly more than 2-fold higher on the coated Matriderm (35.7 ± 1.7%; *p* < 0.0001) than on the other three carriers, which had similarly low ratios (15.0 ± 1.1% for coated PCL/PLA, 13.9 ± 1.2% for uncoated PCL/PLA, and 13.7 ± 0.9% for uncoated Matriderm). Proliferation was balanced for all coated and uncoated carriers without any significant differences (20.1 ± 1.7%, 23.0 ± 2.6%, 23.5 ± 2.2%, 23.0 ± 1.9%) (Figures 5A–C). On day 8, uncoated Matriderm had the lowest apoptosis ratio of all carrier types (19.7 ± 2.4%; *p* = 0.0–0.02). Coated Matriderm had the highest apoptosis ratio (33.7 ± 1.2%) but with only narrow differences between coated PCL/PLA (33.3 ± 2.0%) and uncoated PCL/PLA (26.9 ± 1.8%). Uncoated Matriderm had the highest percentage of proliferative cells (40.9 ± 4.8%), and coated Matriderm had the lowest proliferation (4.9 ± 0.7%; *p* < 0.0001) (Figures 5D–F).

### Mouse Wound Model

**Figure 6:**
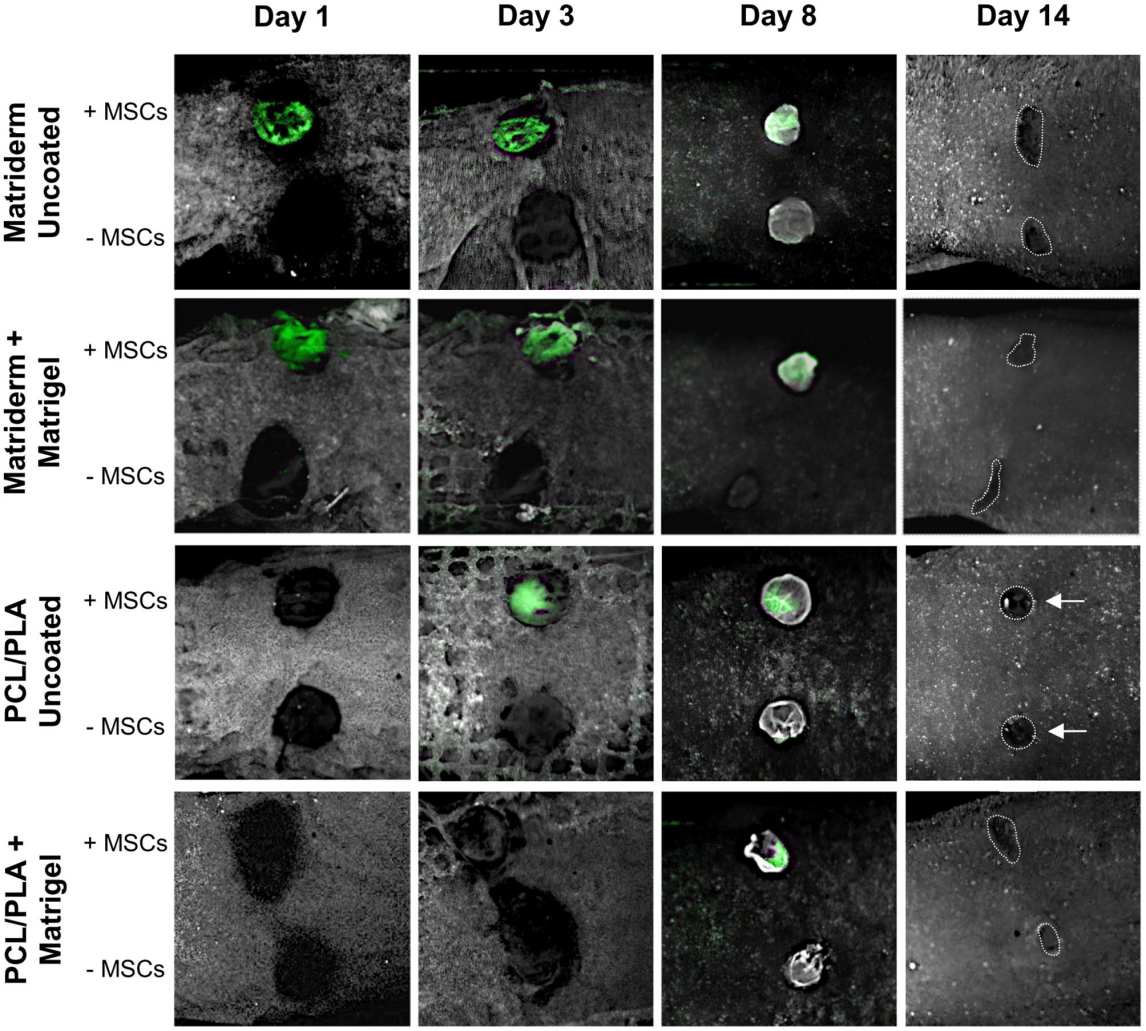
Cell tracking in hAMSC-treated wounds. Carriers cultured with CellTracker-green-labeled hAMSCs or without cells (control) were applied to mouse wounds and monitored using the CRi Maestro *in vivo imaging* system on days 1, 3, 8, and 14. The dotted line indicates the wound margins at day 14. Arrows indicate circular healing.

We performed a laser-scanning microscopical analysis of the carriers cultured for 3 days with CellTracker-green-labeled hAMSCs, which yielded a successful cell population of fluorescent cells (data not shown). Next, the cultured carriers or carriers without cells (control) were applied to the mouse wounds and monitored *in vivo* using the CRi Maestro optical imaging system (Figure 6). On the day of application (day 1), green-fluorescent hAMSCs were detected in the wounds treated with coated and uncoated Matriderm but not in the wounds with coated and uncoated PCL/PLA. On day 3, fluorescent hAMSCs were strongly visible on the uncoated PCL/PLA, whereas the intensity of the fluorescence of hAMSCs in the Matriderm carriers slightly decreased. On day 8, hAMSCs became visible in the coated PCL/PLA, but the fluorescence signal in all hAMSCs-treated carrier types was weak. By day 14, no signals were detected. Wounds treated with carriers without hAMSCs never showed fluorescence.

Until day 8, all wounds had a round appearance, indicating minimal contraction, including the untreated wounds with Tegaderm only (no carriers, no hAMSCs, data not shown). On day 14, all wounds had an irregular appearance except for those treated with uncoated PCL/PLA with and without hAMSCs, which maintained a strikingly round appearance (Figure 6).

**Figure 7:**
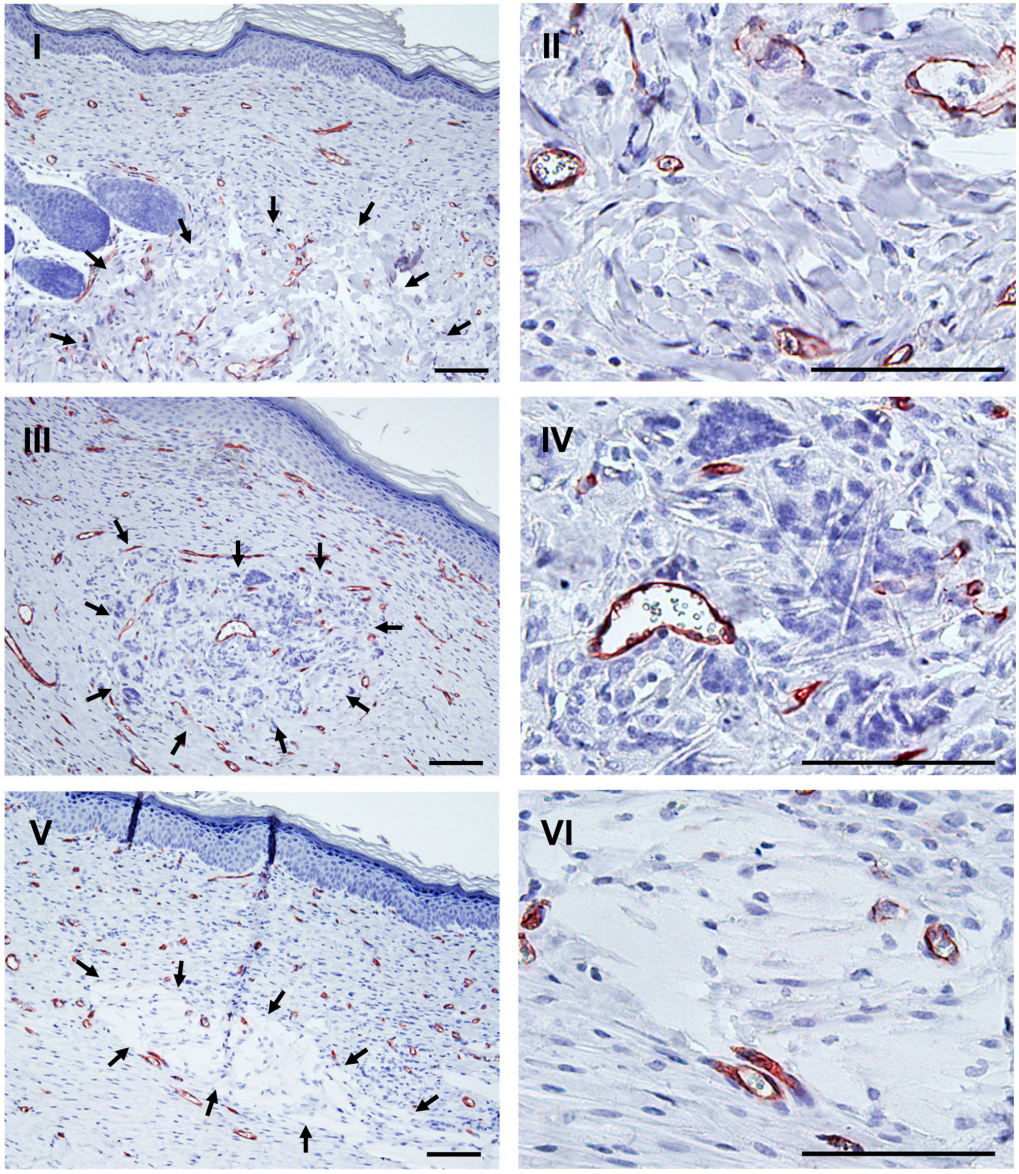
Integration of carrier material into mouse wounds after 14 days of wound healing. Histological evidence of remnants of Matriderm **(I,II)**, PCL/PLA **(III,IV)**, and Matrigel **(V,VI)** in the wound biopsies at low and high magnification, respectively. Remnants are encircled by arrows. Cells were visualized using blue-stained nuclei and capillaries marked by anti-CD31 (brown color). Scale bars = 100 μm.

Histological analysis at 14 days post injury showed that all wounds were covered by a thin epidermal layer and closed (Figure 7). Macroscopical photographs revealed that the wound beds were visible through the thin epidermal layer. Thus, the wound shape could be observed, as documented in Figure 6. All carrier types were well-integrated into the wounds. Fragments of Matriderm (Figures 7I,II), PCL/PLA (Figures 7III,IV), and Matrigel (Figures 7V,VI) were detectable in the dermis. Murine blood vessels stained by anti-mouse CD31 and connective tissue cells could be observed in all degrading materials. The immature dermal layers were still in the regeneration process and therefore not completely rebuilt but enriched by numerous connective tissue cells and blood vessels (Figure 7). We found few vimentin-positive cells and no Ki-67 positive human cells in the tissue sections of the wound biopsies (data not shown).

## Discussion

Biologic skin substitutes are used to treat acute and chronic wounds. These substitutes include skin cells, biopolymer scaffolds, and their combinations (Boyce and Lalley, 2018). The direct injection of cells can be highly inefficient, however, because it incurs substantial cell death due to shear forces inside the syringe (Burdick et al., 2016). Thus, we aimed to develop a hAMSC-seeded skin substitute that keeps treatment cells in the wound area. Polymeric biomaterials can protect MSCs and thus improve their survival and paracrine activity, which has beneficial effects for wound healing (Dash et al., 2018). We created a promising epidermal-like PCL/PLA carrier for hAMSCs using electrospinning. We then compared its properties with Matriderm, a carrier we had already successfully proven in a mouse model of wound healing (Tuca et al., 2016; Ertl et al., 2018). Matriderm and PCL/PLA scaffolds coated with or without Matrigel were seeded with hAMSCs and analyzed for their ease of handling and the quantitative and qualitative adherence of the cells on the scaffolds. Abidance of the hAMSCs to the wounds was investigated using *in vivo* imaging. The carriers' effects on wound appearance also were analyzed. We found that PCL/PLA was the most suitable cell application material for wound healing.

Electrospinning is a versatile method for the rapid and cost-effective production of micro- and nanofibers. Electrospun fibers closely mimic the extracellular matrix structure of native tissue and have desirable properties for wound dressing (Szentivanyi et al., 2011; Mulholland, 2020). By modifying the polymer composition and total polymer concentration, we fabricated two-layer electrospun scaffolds with tailored microstructures and fiber diameters that could be adjusted to the requirements of individual cell types.

Recently, we developed electrospun scaffolds consisting of an inner layer of PCL/PLA (100/50 mg/ml) and an outer layer of PCL (200 mg/ml). These scaffolds showed good cell adherence but were thermosensitive and needed a low-temperature preparation method for histological processing (Fuchs et al., 2019). Fuchs et al. showed that the combination of low-melting-point paraffin embedding and pepsin digestion can be used as an antigen retrieval method for histological investigations of thermosensitive specimens. However, various antigen-demasking enzymes (hyaluronidase and proteinase K) did not work in our present experimental settings, and pepsin digested the Matriderm. Thus, antigen retrieval with heat exposure (93°C) was necessary for further immunohistochemical evaluation of cells in our electrospun scaffolds. As PCL has a lower melting point (57°C) than PLA (169.6°C), we blended the two to achieve thermostable grafts.

We then produced two-layer fiber mats consisting of PCL/PLA (100/50 mg/ml) and PCL/PLA (200/100 mg/ml). Solvent-based blending of PCL and PLA led to a multiphase polymer in which the polymer chains were linked via physical bonds but not covalently bonded, as in the case of a co-polymer (Aslan et al., 2000). The result was a composite-like material with characteristics of both polymers. This effect was confirmed by differential scanning calorimetry to assess the melting points of the PCL and PLA fractions in the multiphase polymer. Thus, we successfully created thermostable scaffolds that were appropriate for the application of conventional histological processing methods.

Among the parameters known to influence the fiber diameter of the electrospun scaffolds, choice of polymer and polymer viscosity are the most efficient. Fiber diameter increases with increasing solution viscosity, which depends on polymer concentration. PCL/PLA 200/100 blends resulted in a thicker layer with larger fibers more loosely arranged than the smaller, densely packed fibers in the PCL/PLA 100/50 layer.

We noted several differences in the ease of handling the materials. For example, due to the hydrophobic character of PCL/PLA, a careful dropwise application of the cell suspension was necessary to facilitate soaking the carrier. In contrast, Matriderm absorbed the cell suspension easily due to its highly porous, sponge-like microstructure. As surface properties like wettability or roughness strongly influence cell adhesion, we performed contact angle measurements to evaluate the wettability of the scaffolds. Interestingly, PCL/PLA and Matriderm showed similar contact angles. Our contact angle measurements showed similar wettability of singular structures (fibers of PCL/PLA and Matriderm) rather than of the whole structure. When Matriderm and PCL/PLA were soaked with the cell suspension, our histological analysis showed good cell adherence to both carrier types. Matriderm was more susceptible to coiling, tattering, and degradation than the stiffer PCL/PLA. Most problems with the Matrigel-suspended cells were due to the jelly-like consistency of Matrigel and to performing all cell seeding on ice. Matrigel attached to both PCL/PLA and Matriderm but did not penetrate these materials. It stabilized the fragile Matriderm but did not improve the handling of PCL/PLA.

Using cross-sections of the hAMSC-seeded scaffolds, we studied the cell distribution using immunohistochemical methods. We demonstrated that the cells did not invade the dense-meshed layer but stayed in the wide-meshed layer for incubation periods of 3 days and 8 days. Our histological analysis of hAMSC-seeded carriers showed the highest cell numbers after 3 days of incubation. The best effects were observed for uncoated PCL/PLA, where most cells were located at the apical surface. In coated and uncoated Matriderm carriers, hAMSCs were observed mostly in the first third of the material. Matrigel contains growth factors TGF-β and PDGF (Kleinman and Martin, 2005). It also exerts cell-stabilizing properties that help keep cells inside the gel but outside the carriers. On day 8, hAMSCs migrated deeper into the scaffolds, even in carriers coated with Matrigel, and colonized the apical half of Matriderm and the complete wide-meshed layer of PCL/PLA. Electrospun fibers possess high surface area to volume ratios. With a decreasing fiber diameter, the specific surface of electrospun scaffolds increases. The surface area of the fine-meshed PCL/PLA 100/50 was about 3-fold higher than that of PCL/PLA 200/100. PCL/PLA 100/50 did not allow cell infiltration, but it protected against mechanical stimuli and enabled the exchange of gas and fluids.

The quantitative analysis showed that on day 3, hAMSCs on all carrier types except one had similarly balanced rates of apoptosis and proliferation. Matriderm coated with Matrigel had significantly enhanced apoptotic cells, perhaps because the dense cell layer in Matrigel resulted in a lack of nutrition and space. On day 8, the apoptosis ratio increased for all carrier types. Conspicuously, on densely populated carriers, proliferating cells were mostly located on the topical side of the cell layer, indicating that cells proliferated due to more space and better access to the supplements in the medium. The sparsely populated uncoated Matriderm had the highest percentage of proliferative cells and lowest apoptosis of all carrier types. Therefore, an experiment with lower seeding density could be used to assess long-term hAMSC viability *in vitro*. Overall, the *in vitro* data showed that compared to 3 days of incubation in cell-seeded carriers, 8 days of incubation did not improve the properties of hAMSCs.

We thus used the cell-seeded carriers cultured for 3 days *in vitro* in our mouse model of wound healing. We applied the two-layered PCL/PLA scaffold such that the wide-meshed (cell-populated) side directly faced the wound bed and served as the basal layer and the dense-meshed (cell-free) layer served as the apical layer. We tracked fluorescent-labeled hAMSCs in the mice using *in vivo* imaging on day 1 of application and again on days 3, 8, and 14. All carriers stayed in the wound regions without suturing. On the coated and uncoated Matriderm carriers, hAMSCs had strong fluorescent signals on days 1 and 3 and weaker signals on day 8. In contrast, hAMSCs were not detectable on PCL/PLA carriers on day 1, then signals were observed on day 3 on uncoated PCL/PLA and on day 8 on coated PCL/PLA. We suspect that the apical PCL/PLA layer served as a barrier that prevented our *in vivo* imaging system from visualizing the fluorescent cells in the underlying layer. The eventual degradation of the superficial dense layer likely helped to reveal the fluorescent cells in the underlying wide-meshed layer. Thus, Matrigel seemed to delay the degradation of the PCL/PLA layer. On day 8, we detected hAMSCs on all carrier types within the wound area, indicating abidance of the hAMSCs to the wounds and no migration into other tissue. We concluded that because the hAMSCs were incubated on the carriers before applying them to the wounds, they were already adherent and could be fixed in the wound area. Adherence of cells to wounds promotes longer wound healing, and even dead hAMSCs release curative factors (Farhadihosseinabadi et al., 2018). We cannot exclude the possibility that the persistence of the fluorescent signal we observed up to day 8 could have come from phagocytosed cells in the wound bed.

On day 14, our histological analysis showed that all wounds were closed and covered by a newly formed thin epidermal layer and that all carriers had mostly degraded. Remnants of PCL/PLA, Matriderm, and Matrigel were found beyond the epidermis, and they were well-integrated into the dermal layer of the wound area. This biodegradability allowed space for cell expansion, cell migration, and neovascularization. The observed loss of cell tracker signals could be due to tissue restricting access to the fluorescence signal or to signal dilution caused by cell proliferation, migration, or death. Kim et al. (2012) showed that hAMSCs engrafted into the wound area and directly participated in re-epithelialization via trans-differentiation into keratinocytes. As we found few vimentin-positive cells and no Ki-67 positive (proliferative) human cells in the tissue sections of the wound biopsies on day 14 post injury, we suggest that the hAMSCs migrated out of the wound area or trans-differentiated into skin cells. This assumption is supported by our previous study on a Matrigel-plug angiogenesis model, which showed a successful integration of human cells into the murine blood vessel system (Kinzer et al., 2014). The wound bed was macroscopically visible through the thin epidermal layer. The dermal layer was still in the regeneration process and therefore not completely rebuilt. Strikingly, only wounds treated with PCL/PLA without Matrigel still had a round appearance on day 14, regardless of whether hAMSCs were applied, whereas the other treated and untreated wounds had irregular shapes. We ascribe this effect to the stiff material of our PCL/PLA scaffolds, which may have promoted an anti-contractive effect even after the carriers partly degraded. Interestingly, Lee et al. (2014) observed a higher wound contraction among Matriderm-treated wounds than wounds treated with an electrospun silk fibroin nanofiber matrix. PCL/PLA thus may offer similar or better wound healing benefits, which may have significant implications for prospective wound healing experiments in rodents.

Due to the low cost and wide availability of appropriate antibodies, most lab-based *in vivo* assessments of wound closure and development are performed in rodents. However, rodent wounds close due to contraction of the musculus panniculus carnosus, which is virtually non-existent in humans. This physiological difference creates difficulties in replicating the wound closure processes of human skin (Wang et al., 2013; Hu et al., 2018b). To overcome this problem, a short-term (e.g., 8-day) experiment in which no obvious contraction occurs is preferable (Tuca et al., 2016; Ertl et al., 2018). Longer experiments have used splinting wound models (Wang et al., 2013) or direct suturing of scaffolds to the edges of the experimental wounds (Anjum et al., 2017), but both of these methods carry risks of inflammation and surgical site infection (He et al., 2009; Mashhadi and Loh, 2011; Mulholland, 2020). The application of PCL/PLA without suturing could enable uncomplicated experiments on rodents and thus open new therapeutic approaches.

Novel and effective anti-scarring treatments are clinically absent and highly warranted because scarring is a huge healthcare burden worldwide. It can lead to many adverse side effects, such as reduced mobility, compromised organ function, functional disabilities, and psychological stress. In hypertrophic scars, myofibroblasts can cause persistent contraction that leads to skin dysfunction (Ehrlich et al., 1994). PCL/PLA scaffolds may be useful clinical tools to avoid such scarring.

Previously, we demonstrated the beneficial effects of hAMSCs on wound healing by showing that hAMSCs induce enhanced neovascularization and accelerated wound closure (Tuca et al., 2016; Ertl et al., 2018). In our present study, we aimed to find the most promising carrier for hAMSCs in a mouse model of wound healing. Both PCL/PLA and Matriderm led to good cell adherence in the wound area until day 8. Based on our *in vitro* results and the performance of the carriers in our animal experiment, we found the PCL/PLA carrier to be the most suitable for future testing in other wound healing models. In a next step, we will evaluate the effect of hAMSC-seeded PCL/PLA carriers on wound healing and their potential anti-scarring effects in larger animal groups.

## Conclusion

Both Matriderm and PCL/PLA scaffolds are suitable as carriers for mesenchymal stromal cells. Short incubations and seeding without Matrigel provided the best environment for these cells. Compared to Matriderm, PCL/PLA scaffolds yielded a higher number of attached cells and more favorable growing conditions for hAMSCs, and they exerted anti-contractive properties in the wound area. Our PCL/PLA scaffold had a two-layer structure: a wide-meshed (cell-populated) layer facing the wound bed and serving as the basal layer and a dense-meshed (cell-free) layer serving as an apical barrier of small fibers protecting the cells from the outside environment. This PCL/PLA scaffold offers a promising epidermis substitute.

## Data Availability Statement

The raw data supporting the conclusions of this article will be made available by the authors, without undue reservation.

## Ethics Statement

The studies involving human participants were reviewed and approved by Ethical Committee of the Medical University of Graz. The patients/participants provided their written informed consent to participate in this study. The animal study was reviewed and approved by Animal Care and Use Committee in Vienna.

## Author Contributions

EV, MM, and IL-O designed the experiments, analyzed the data, prepared the figures, and wrote the manuscript. MM fabricated the electrospun scaffolds. EV and MM conducted the majority of the experiments. MP, AH, A-CT, and L-PK were responsible for the animal experiments. MS performed the histological processing and analysis. SAW and BR conceived and performed the *in vivo* optical imaging. IL-O supervised the project. All authors discussed the results and revised the manuscript.

## Conflict of Interest

The authors declare that the research was conducted in the absence of any commercial or financial relationships that could be construed as a potential conflict of interest.

## Footnotes

^1^https://www.prnewswire.com/news-releases/global-scar-treatment-market-2013-2018--2023---rise-in-online-retailing-of-scar-treatment-products-300698219.html
